# Caste, gender, and everyday resistance: a cultural psychological reading of Bama’s fiction

**DOI:** 10.3389/fpsyg.2026.1911862

**Published:** 2026-10-08

**Authors:** S. Prabakar, M. Ananthi

**Affiliations:** Department of Social Sciences, School of Social Sciences and Languages, Vellore Institute of Technology, Vellore, India

**Keywords:** Bama, caste identity, cultural psychology, Dalit literature, gender, postcolonial psychology, resistance, social exclusion

## Abstract

This study offers a cultural psychological reading of how caste-based oppression and everyday resistance to it are represented in contemporary Tamil Dalit fiction. Using Bama’s short story collection *The Ichi Tree Monkey* (2021) as the primary text, this paper applies a cultural psychological framework to analyse 15 stories across the themes of caste identity, gender, labour, education denial, and resistance. The study draws on postcolonial thought and community psychology to show that Bama’s characters move from conditioned submission to conscious self-assertion, a shift whose underlying logic—quiet and cumulative resistance under conditions where open confrontation is unsafe—resonates with frameworks developed to describe resistance to discrimination based on race and gender in other national contexts, even though caste itself remains a system specific to South Asia. The study also pays close attention to the position of Dalit women within this system, since their experience of caste cannot be separated from their experience of gender. Findings show that resistance in Bama’s fiction is not always dramatic or political. Far more often it is quiet, daily, and deeply human, appearing in a kept bus seat, a stolen laugh, or a refusal to use a respectful title for someone who has not earned respect. This paper contributes to Dalit literary and cultural psychological scholarship in three specific ways: by applying Scott’s theory of everyday resistance and hidden and public transcripts to a body of Dalit fiction for the first time, by demonstrating through close reading how caste and gender compound one another in the specific forms of resistance available to Dalit men and Dalit women respectively, and by arguing methodologically that sustained literary analysis of a Dalit-authored text can itself serve as a legitimate and rigorous method within cultural psychology, rather than as illustration for arguments developed elsewhere.

## 1 Introduction

Caste is not only a social system that decides who marries whom or who eats with whom. It is also a psychological environment that surrounds a person from the moment they are born and never fully leaves them, even when they move to a new place or gain a new qualification. It shapes what people believe they deserve in life, what they learn to fear without ever being told to fear it, and what they quietly assume is simply not meant for someone like them. A child raised inside this environment absorbs its rules long before they can explain what those rules are or where they came from, and this absorption is what makes caste so difficult to study through statistics alone. Numbers can tell us how many children drop out of school or how many families remain landless across generations, but they cannot easily show us what it feels like, from the inside, to be told every day in small and large ways that you come last.

Bama is a Tamil Dalit author whose short story collection *The Ichi Tree Monkey* (2021) captures this inside view with rare honesty and without any need for exaggeration. Published by Speaking Tiger Books, the collection contains 15 stories set in rural Tamil Nadu, and each one is built from the texture of ordinary village life rather than from dramatic, headline-grabbing events (Bama, 2021). The people in these stories are farmers, domestic workers, cattle grazers, schoolchildren, and women who cut grass on the edges of someone else’s land because their own families have nothing left to eat. Bama does not introduce them as victims who need to be explained to the reader. She introduces them as people first, with names, habits, jokes, and quiet pride, and only afterwards do we see the weight that caste places on their daily lives.

What makes Bama’s work especially important for cultural psychology is the fact that her characters are never simply passive recipients of suffering. They respond. They push back, sometimes loudly and sometimes so quietly that an outside reader could almost miss it if they were not paying close attention. They find small, stubborn, and often very clever ways to hold on to a sense of dignity, even inside a system that was built specifically to deny them that dignity. This paper reads those responses through a cultural psychological lens, treating the stories not as decoration on top of a sociological argument but as a primary source of insight in their own right, in the same way a researcher might treat an interview transcript or a piece of ethnographic field writing.

Haider (2015) argues that Bama’s narrative style blends a deeply personal voice with the collective memory of her community, and this blending is what gives her fiction the unusual status of being both a literary work and a kind of social record at the same time. Arivu Mani (2024) documents a similar pattern of resistance and resilience in Bama’s novel Vanmam, suggesting that the psychological dynamic traced in this paper is not confined to a single collection but recurs across Bama’s wider body of work. This paper builds directly on that observation by asking a focused question. What do Bama’s characters reveal to us about the psychological experience of living under caste, and equally important, what do they reveal about the human capacity to resist that experience even when resistance carries real risk? The remainder of this paper attempts to answer that question by reading the 15 stories thematically, in the groupings set out in Section 6, before drawing the threads together in the conclusion.

## 2 Research questions and objectives

This study is organised around three central research questions, each addressed across the story analysis sections and revisited again in the discussion. These questions were chosen because they reflect the most consistent and recurring concerns that emerge from a close reading of all 15 stories in the collection, read in full rather than through a small sample selected for convenience, and because together they allow the paper to move from individual character experience toward a broader cultural psychological argument.

RQ1: How do Dalit characters in Bama’s stories experience caste-based oppression at a psychological level, in terms of fear, self-worth, and a sense of what is possible for them in life? Answering this question requires identifying and describing how caste functions as a psychological system within Bama’s fictional world, shaping behaviour, speech, and self-perception long before it shapes anything material.

RQ2: What forms of resistance do these characters use in response to that oppression, and what do those particular forms reveal about their sense of identity and personal agency?

RQ3: How do caste and gender intersect to shape the experiences of Dalit women specifically, and how does this intersection differ from the experiences of Dalit men in the same stories? Answering this question requires examining carefully the intersection of caste and gender in shaping the experiences of Dalit women, since their position cannot be reduced to caste alone or gender alone.

Underlying these three questions is a broader methodological objective: to demonstrate, through sustained close reading, that literary fiction written from within a marginalised community can serve as a legitimate and valuable source of cultural psychological knowledge, rather than as illustration of arguments developed elsewhere.

## 3 Methodology

This study uses qualitative literary analysis as its primary method, a well-established approach within cultural psychology in which fictional texts are read closely as cultural documents that reveal something of the psychological realities experienced by the communities they represent (Jaiswal, 2023). Literary analysis of this kind does not attempt to prove a hypothesis through measurement in the way a laboratory study might. Instead, it works through close, repeated, and careful reading of the text, paying attention to what characters say and do, what they are forced to accept, and what small moments of refusal or assertion appear even within otherwise difficult circumstances. The strength of this method lies in its ability to capture nuance, contradiction, and lived texture in a way that survey data alone often cannot.

The primary text for this study is *The Ichi Tree Monkey* (Bama, 2021), a collection of 15 short stories published by Speaking Tiger Books. The edition used for this study is the first Speaking Tiger paperback edition (2021, 160 pages, ISBN 978-93-89231-48-9), cited here to fix the specific pagination and printing referenced throughout the story analysis, since page counts for this title differ across some online listings. The collection was originally written in Tamil and is read here in its English translation by N. Ravi Shanker, a factor that necessarily shapes the present analysis, since questions of tone, dialect, and the specific connotation of caste-inflected address, central to a story such as Annachi, are mediated by the translator’s choices rather than accessed directly from the Tamil original. All 15 stories were read in full and included in the analysis, with none excluded or treated as secondary, because the study aims to represent the full range and texture of the collection rather than a partial selection chosen for convenience. Secondary sources used alongside the primary text include peer-reviewed scholarship in Dalit studies, postcolonial theory, gender studies, agrarian labour studies, and community or cultural psychology, all of which are listed in full in the reference section at the end of this paper.

The analytical framework draws on four complementary ideas from existing psychological and social science literature. The first is the cultural psychological concept of identity within context, which holds that a person’s sense of self cannot be properly understood separately from the cultural, social, and material environment in which that person lives their daily life (Pal, 2015). This view is consistent with a broader tradition within cultural psychology holding that selfhood is fundamentally shaped by the cultural and social meanings available to a person, from Shweder’s (1991) account of psyche and culture as mutually constitutive, to Markus and Kitayama’s (1991) demonstration that even basic self-construal varies systematically across cultural contexts, to Valsiner’s (2007) and Hermans’s (2001) related arguments that the self is populated by multiple, culturally positioned voices rather than existing as a single fixed entity. The second is the postcolonial psychological understanding of resistance, which holds that communities living under long term structural oppression develop their own internal strategies, both small and large, to survive that oppression and to assert a sense of selfhood within it, even when open political resistance is not possible or safe (Bikshapathi, 2021). Fanon’s (2008) account of how colonised subjects internalise the coloniser’s gaze before they can begin to refuse it, and Nandy’s (1983) related argument that colonialism produces its own distinct psychology of self doubt in both the colonised and the coloniser, extend this understanding beyond the specifically Tamil context and situate Bama’s characters within a broader postcolonial psychological literature on subjecthood under domination. Bhatia’s (2018) contemporary work on decolonising psychology and Teo’s (2010) critique of epistemological violence within the empirical social sciences further support the methodological premise of this paper, that a psychology developed primarily within Western, laboratory based traditions requires deliberate supplementation with culturally situated methods, such as sustained literary analysis, if it is to adequately represent the psychological realities of formerly colonised and caste-subordinated communities. The third is Scott’s (1985, 1990) account of everyday resistance among subordinate groups, which holds that open rebellion is rare precisely because it is dangerous, and that resistance therefore tends to take the form of small, low-visibility acts of non-compliance that stop short of direct confrontation while still refusing full submission. Central to this account is the distinction between the public transcript, the outwardly compliant and deferential behaviour that subordinate groups display in the presence of those who dominate them, and the hidden transcript, the critique, mockery, and plain speech that the same groups reserve for spaces where power is not watching. This distinction offers a precise vocabulary for a pattern that recurs throughout Bama’s fiction, in which characters observe every outward convention of caste deference until a specific moment in which the hidden transcript breaks through into the open. The fourth is Bruner’s (1990) account of narrative as a primary vehicle through which people construct and make sense of the self, which holds that identity is not simply reported by stories but actively built through them, since it is by narrating a life, or having it narrated on one’s behalf, that a person comes to understand what that life has meant. This provides the specific methodological warrant for treating Bama’s stories as psychological evidence in their own right rather than as illustration, since if selfhood is itself narratively constructed, then a carefully constructed narrative is a legitimate site at which to study it.

Working from these two ideas together, each of the 15 stories was analysed along three lines of inquiry, with gender integrated systematically into each rather than treated as an optional addition, consistent with RQ3’s concern with intersectionality. First, how do the characters in the story experience caste, and, for women characters specifically, how does gender based oppression compound that experience in their immediate, lived circumstances? Second, how do they respond to that oppression, whether through open defiance, quiet endurance, clever subversion, or simple survival, and whether the specific form of response available to them differs systematically between Dalit men and Dalit women. Third, what does their particular response reveal about questions of identity, agency, and the broader psychology of communities living under sustained social exclusion? This three-part structure is applied consistently across every story, producing a short written note for each of the 15 stories that records its answers to these three questions.

The six cross-cutting themes presented in Table 1 and developed in Section 6 were generated from these 15 story-level notes through a process of constant comparison, the standard procedure for moving from coded observations to themes in qualitative thematic analysis (Braun and Clarke, 2006). Each story’s notes were read against every other story’s notes to identify observations that recurred in substantially similar form across multiple, independently written stories, rather than being unique to a single plot. A recurring observation was provisionally treated as a candidate theme once it appeared in this way across at least three stories; candidates that met this threshold were then checked against the theoretical framework set out above, retaining only those that could be connected to an existing concept in that framework (for example, the recurring observation that caste is enforced through everyday spatial and linguistic detail was retained as a theme because it connects directly to Pal’s (2015), account of social exclusion and self-perception, and to Scott’s (1985, 1990), account of the public transcript). This checking step was intended to guard against mistaking a merely descriptive similarity across stories for an analytically meaningful pattern. The six themes that survived this process, caste as everyday architecture, labour and debt as structural control, the systematic denial of education, the compounding of caste and gender, small-scale individual resistance, and collective community resilience, are not the only patterns visible across the collection, but they were the ones that recurred most consistently and connected most directly to the paper’s theoretical framework.

**Table 1:** Cross-cutting psychological themes and supporting stories.

Theme	Key stories	Psychological concept	Scholarly support
Caste as daily architecture	Pongal, Annachi, Ponnuthayi	Social exclusion and self-perception	Pal (2015)
Labour and debt as control	Rich Girl, Old Man, Empty Nest	Structural dependency and precarity	Soundararajan et al. (2023), Sinha (2024)
Education systematically denied	Rich Girl, Freedom, Half Sari	Foreclosed possibility and identity	Kumar (2025), Human Rights Watch (2015)
Gender and double marginalisation	Chilli Powder, Half Sari, Single	Intersectionality of caste and gender	Mangat and Gill (2024), Thiyagarajan (2024)
Resistance, small and daily	Annachi, Single, Those Days	Agency, identity reclamation	Pan (2018), Bikshapathi (2021)
Collective community resilience	Those Days, Ancharamanippoo Tree	Conscientisation, solidarity	Bikshapathi (2021)

## 4 Historical and cultural context

The Paraiyar and Pallar communities who appear throughout Bama’s fiction belong to Scheduled Caste groups in Tamil Nadu whose marginalisation has very deep historical roots, stretching back long before independent India even existed as a political entity. For many centuries, these communities were tied closely to agricultural labour on land owned by upper caste landlords, denied any meaningful access to formal education, expected to enter homes through back doors rather than front entrances, and required to show visible, public deference whenever an upper caste person was nearby, regardless of that person’s actual age or standing (Bikshapathi, 2021). These were not occasional humiliations reserved for special occasions. They were the basic, unremarkable texture of daily interaction, repeated so often that they became, for many, simply how the world worked.

The Self Respect Movement, founded by Periyar E. V. Ramasamy in Tamil Nadu during the 1920s, began to challenge this deeply entrenched social order in a sustained and organised way. It taught Dalit communities, often for the first time in an explicit and public form, that their subordination was not a natural fact about the world or about themselves, but rather a social arrangement constructed and maintained by those who benefited from it, and that such an arrangement could therefore be questioned, resisted, and eventually dismantled (Bikshapathi, 2021). This was a profound psychological shift as much as a political one, because it offered, for the first time to many ordinary people, a language in which their experience of unfairness could be named as unfairness rather than simply accepted as fate. B. R. Ambedkar’s contemporaneous work at the national level gave this same psychological shift a further, more systematic foundation, arguing in Annihilation of Caste (Ambedkar, 2014) that caste could not be reformed piecemeal because it was sustained not by law but by a shared religious and social sanction internalised by oppressor and oppressed alike, and that genuine change therefore required nothing less than a fundamental transformation of consciousness on both sides. Where Periyar’s movement worked through the specific cultural and linguistic terrain of Tamil Nadu, Ambedkar’s argument supplied the broader conceptual claim that caste is above all a state of mind before it is a social arrangement, which underlies the psychological reading of resistance offered throughout this paper.

This historical shift between an older world of accepted subordination and a newer world of questioning and assertion is visible throughout Bama’s stories, almost as a structural pattern running beneath the surface of the individual plots. Older characters in the collection tend to accept the system they were born into, often defending it to their own children when those children begin to question it. Younger, more often educated characters tend to refuse the same system, sometimes quietly and sometimes with open defiance. This generational gap between submission and resistance is one of the most consistent threads running through the entire collection, and it gives the 15 stories, read together, a kind of cumulative argument about social change that no single story could make on its own.

The economic conditions that Bama describes throughout the collection remain genuinely difficult, and this is not simply a matter of historical memory, but an ongoing reality documented by contemporary researchers. Sinha (2024) documents how Dalit women agricultural workers in India continue to face land denial, wage discrimination, and exclusion from formal labour protections that other workers can rely upon. Soundararajan et al. (2023) show that social capital within rural labour markets is largely controlled by upper caste intermediaries, leaving Dalit workers with very little institutional recourse when they are harmed in the course of their work. Mangat and Gill (2024) further show that Dalit women who attempt to speak out against violence frequently face silencing pressures from both caste-based and gender-based systems operating together rather than separately. Bama’s fiction gives these structural realities a human face and a human voice, and as Jaiswal (2023) argues, *The Ichi Tree Monkey* can be understood as an act of cultural reclamation, deliberately bringing community stories that had been pushed to the margins back into full literary view, where they can be read, discussed, and taken seriously by audiences far beyond the village settings in which they take place.

## 5 Story overview table

Table 2 below provides a structured summary of all 15 stories analysed in this paper, listing the central characters, the core psychological theme that the story foregrounds, and the particular form of resistance, if any, that appears within it. This table is intended as a navigational aid for the reader and as a way of presenting the collection’s overall shape before the thematic analysis, organised around cross-cutting patterns rather than individual stories, begins in the following section.

**Table 2:** Summary of stories and key themes.

Story	Central character(s)	Core psychological theme	Form of resistance
Pongal	Madasami, Esakkimuthu	Conditioned submission, ritual inequality	Realisation, generational break
Annachi	Ammasi, Chandrasekhar	Linguistic hierarchy, identity assertion	Refusing deference, equal address
Harum Scarum Saar	Teacher, Dalit children	Authority as unpredictable threat	Silence as a survival strategy
Chilli Powder	Pachayamma, Gangamma	Double marginalisation, survival logic	Continuing to work despite violence
Rich Girl	Kaliyamma, Ramayi, Muthu	Intergenerational harm, education denial	Endurance, naive hope
Those Days	Maruthappan	Conscientisation, collective action	Public confrontation, refusal to settle
Ponnuthayi	Ponnuthayi	Internalised servitude, invisible labour	Nonvisible, absence itself is the message
Half Sari	Chellakkili, Irulappan	Betrayal of trust, exploitation of hope	None, betrayal forecloses agency
Freedom	Arayi, Subramani	Chronic stress, suppressed selfhood	Song and laughter, longing for education
Old Man and a Buffalo	Malandi Thatha	Resilience without illusion, bonded debt	Fearlessness, refusing internalisation
The Ichi Tree Monkey	The monkey, allegorical	Folk survival wisdom	Cunning, evasion of capture
Stereotype	Kalaivani, young boy	Premature adult identity, gendered burden	Declaration of self, I am a man
Single	Illamalli	Social stigma, gender-based labelling	Physical resistance, reclaiming identity
The Ancharamanippoo Tree	Community	Cultural reclamation, sacred space	Gathering, asserting community rights
Empty Nest	Elderly couple	Displacement, agrarian precarity	Memory, dignity in solitude

## 6 Thematic analysis

Rather than proceeding story by story, the analysis below is organised around six cross-cutting psychological themes that recur across the collection, summarised in Table 1 and developed in turn in the six subsections that follow.

### 6.1 Caste as a totalizing environment: space, speech, and institutional authority

Across the collection, caste rarely announces itself through a single dramatic act of violence. It operates instead through the patient repetition of small spatial, linguistic, and institutional inequalities, repeated so consistently that they eventually come to feel like the natural order of things rather than an imposed and unjust arrangement. Pongal, Annachi, Harum Scarum Saar, and The Ancharamanippoo Tree, read together rather than in isolation, show this same underlying mechanism operating across four different registers of everyday life: the household, the road, the classroom, and the sacred grove.

Spatial hierarchy is the clearest instance of the pattern. In Pongal, Madasami prepares, without exception, an elaborate yearly tribute for his landlord Ramasami Ayya: pumpkin, sugarcane, bananas, and rice, representing a significant share of what his small landholding produces, in exchange for a single 10-rupee towel. He is directed, as always, to the backyard near the cowshed rather than the front entrance, and he accepts this without protest, as he has every year before. It is only when the landlord fails even to provide the customary towel that something inside him finally shifts, and he understands in concrete rather than abstract terms what his son Esakkimuthu had been arguing all along. The instruction to use the backyard rather than the front door is significant here because spatial hierarchy of this kind, who is permitted to enter through which door, is one of the most enduring and effective ways in which caste communicates a person’s supposed place in the social order (Pal, 2015). There is also something troubling in the underlying logic Bama exposes without ever stating it directly: the landlord eats what Madasami’s land produces and benefits from his labour across the year yet will not allow him through the front door of his own house. Whatever is supposedly impure about Madasami and his community is apparently not impure enough to prevent the landlord from consuming what they grow, a contradiction that sits at the very heart of caste-based thinking (Bikshapathi, 2021).

Where Pongal shows spatial hierarchy, Annachi shows linguistic hierarchy operating with the same underlying logic. Ammasi, Madasami’s son, keeps his seat on a crowded bus when his landlord Chandrasekhar boards and clearly expects him to stand, breaking with a long-standing custom under which lower caste people rise immediately whenever a landlord appears. He then addresses the landlord as Annachi, elder brother, the very term he would use for a Pallar drain cleaner he knows from the village, an act that is disrespectful not because the word itself is rude but because it deliberately removes the linguistic distance the caste hierarchy depends on to function smoothly. Read through Scott’s (1990) distinction between public and hidden transcripts, the episode is doubly significant. Refusing to stand and using Annachi as a term of address are breaches of the public transcript, the visible script of deference caste ordinarily requires. His later remark to his own father, comparing all men regardless of caste to the indistinguishable waste of a single donkey, belongs instead to the hidden transcript, the blunt, levelling critique lower caste communities ordinarily reserve for spaces the landlord cannot hear. Jaya (2026) observes that in Bama’s fiction more broadly, language itself, specifically who is permitted to address whom and by what title, functions as a constant and active site of caste negotiation rather than a neutral backdrop, and the historical background traced in Section 4 confirms that the caste hierarchy Bama’s characters inhabit depended for its everyday functioning on linguistic exclusions as much as physical ones (Bikshapathi, 2021).

Harum Scarum Saar relocates this same dynamic into the classroom. A teacher whose behaviour swings unpredictably between warmth and disproportionate punishment leaves the Dalit children in his class in a more or less constant state of low-level anxiety about which version of him they will encounter on a given day, mirroring in a smaller and more contained setting the much larger power landlords hold over adult labourers elsewhere in the collection. What the children absorb, alongside whatever is being taught that day, is the broader lesson that authority can be unpredictable and that the safest available response is cautious, watchful silence. This connects to documented patterns of discriminatory treatment toward Dalit children in Indian schools, including corporal punishment, informal segregation, and the assignment of degrading tasks specifically to lower caste students (Human Rights Watch, 2015). Bama shows no outright physical violence here, only uncertainty and quiet fear, but she treats that psychological unpredictability as its own distinct form of harm, capable of shaping a child’s relationship to authority for the whole of their later life (Kumar, 2025).

The Ancharamanippoo Tree extends the same logic from the individual to the community. When upper caste landlords attempt to restrict access to a sacred tree that lower caste communities have long used for festivals and gatherings, explicitly claiming ownership over both the tree and the surrounding land, the community firmly resists, insisting on its established right to maintain its own sacred space. Bikshapathi (2021) traces how the Self Respect Movement encouraged Dalit communities across Tamil Nadu to reclaim their own religious and cultural practices from beneath the dominance of Brahminical tradition, and this story demonstrates that reclamation is not an abstract ideological position but as a concrete action: a community gathering around a tree that has always belonged to them. Read alongside Pongal’s backyard and Annachi’s bus seat, the contested tree confirms that the boundary Bama’s fiction returns to again and again—who may occupy which space, on whose terms- is never merely physical. It is the mechanism through which caste communicates and reproduces itself, one ordinary day at a time, and it is precisely this totalizing, cumulative quality, rather than any single dramatic confrontation, that Pal’s (2015) account of social exclusion and self-perception helps to name.

### 6.2 Labour, debt, and the economics of caste

Labour in Bama’s fiction is never simply an economic matter. It is, at the same time, a relationship of power between people of unequal social standing, extracted and controlled in ways that leave workers with essentially no recourse when things go wrong, as Old Man and a Buffalo, Rich Girl, and Empty Nest together demonstrate.

Old Man and a Buffalo makes the mechanics of that control explicit through debt. Malandi Thatha, an elderly cattle grazer loved throughout the village, had originally borrowed 200 rupees from the landlord Rajaram Ayya and has since repaid more than a 1,000 rupees in accumulated interest alone, while the original principal has remained completely unchanged. Soundararajan et al. (2023) document precisely this kind of arrangement as a recognised mechanism of labour control across rural India, one that binds workers to landlords through obligations that are structurally almost impossible to discharge. What distinguishes Malandi within the collection is that he understands this trap with complete clarity and refuses, through sheer force of character, to let that understanding diminish his own dignity. Unlike almost everyone else in the village, he meets a landlord’s gaze steadily rather than lowering his eyes, a fearlessness that is not ignorance of how the system works but a deliberate refusal to internalise its logic while remaining fully aware of its effects.

Rich Girl shows what happens when that same structural power is allowed to operate without even Malandi’s margin of personal dignity to soften it. Kaliyamma and Muthukaruppan are daily wage labourers whose landlord refuses to accommodate their infant son at the worksite and offers no alternative, a refusal that eventually forces their daughter Ramayi out of school entirely once the arrangement built to work around it collapses. When the landlord’s bullock later becomes stuck in a flooded pond, he orders Muthu in after it without pausing to consider the danger, and Muthu, unable to refuse a direct order from a landlord regardless of personal risk, drowns retrieving an animal that survives him. The village does not discuss the landlord’s role in this preventable death; it circulates stories of water spirits instead, since it is safer to blame the supernatural than to hold a powerful landlord accountable for his own carelessness. Soundararajan et al. (2023) document how Dalit workers across India typically operate within labour arrangements where accountability rests entirely with upper caste employers, leaving no institutional mechanism through which justice can be pursued, and the landlord’s 1,000 rupee compensation, which Ramayi mispronounces with childlike pride as proof her family is now rich, is Bama’s quiet portrait of exactly that system in operation, a human life valued at less than a month’s household expenses.

Empty Nest closes this thread by showing where the accumulated weight of debt and unaccountable labour ultimately leads away from the village altogether. An elderly couple’s grown children have all moved to cities or other villages, not because they simply wished to move on with their lives but because the village itself has nothing left to offer them, no land of their own to inherit, no economic opportunity beyond manual labour, and no visible future that does not repeat the servitude their parents endured. This pattern of migration is a well-documented consequence of agrarian caste structures that consistently deny young Dalit people any meaningful economic future within their home villages (Sinha, 2024). Read against Malandi’s undischargeable debt and Muthu’s unaccountable death, the empty house the couple are left folding laundry inside is the structural endpoint of the same economic logic, one Bama tells entirely through a mother’s quiet perspective rather than through argument, trusting the reader to supply the connection themselves.

### 6.3 Education denied, futures foreclosed

Education appears throughout the collection as something that is promised, longed for, and then, far more often than not, quietly taken away, and Freedom and Stereotype show two distinct mechanisms by which that foreclosure happens, alongside Ramayi’s education already lost to economic necessity in Rich Girl above.

In Freedom, Arayi and the young boy Subramani both live within a landlord’s household in conditions closely resembling imprisonment, unable to speak freely, rest when needed, or in Arayi’s case, eat until everyone else has finished. Subramani wants desperately to attend school and approaches a neighbourhood teacher directly, asking her to take him away from the household so that he can study, but she is herself afraid of the landlord’s likely reaction and offers no help. Dalit domestic workers across India occupy one of the most legally unprotected categories of labour, frequently bound to employer households through debt and informal obligation without any written contract or meaningful recourse (Soundararajan et al., 2023), and children placed into domestic service of this kind are typically among the first to lose access to schooling altogether, since the needs of the employing household are almost always prioritised over any educational need the child might have (Human Rights Watch, 2015). The one afternoon the landlord and his wife leave the house, Subramani sings, and Arayi laughs, apparently for the first time in a long while, a single unguarded afternoon that represents, for people whose daily lives otherwise offer no meaningful autonomy, very nearly everything available to them.

Stereotype shows education foreclosed by a different route, not an employer’s refusal but poverty and loss compressing childhood itself. Two young brothers arrive at school and cannot stop crying; their father has recently died of long-term alcohol addiction, leaving their mother to support the family alone. When their teacher, Kalaivani, gently tells the older boy, already in the fourth standard, that his mother can protect herself and that his presence at home would not change that, he replies simply that he may be a small child, but he is nonetheless a man. Bama neither celebrates nor criticises this statement; she presents it with sustained attention, understanding that the boy has already absorbed the weight of adult male responsibility before he has had any real opportunity to be a child. Kumar (2025) notes that for many Dalit children, the combined pressures of poverty and social expectation foreclose the extended period of childhood that meaningful education typically requires and assumes, and Bama gives that observation a human face rather than a statistic: a small boy in an ordinary classroom who already sincerely believes himself a man, and who carries that belief forward as a weight he never chose to take on.

Read together with Ramayi’s exit from school in Rich Girl, these three cases show education being foreclosed by three distinct but related mechanisms: an employer’s flat refusal to accommodate a family’s basic needs, a household’s total demand on a child’s time and labour, and a poverty so acute that childhood itself is curtailed before schooling can matter, each of them converging on the same outcome regardless of the specific path that leads there.

### 6.4 Gender and the compounded burden

The stories examined so far establish caste as a totalizing daily environment and labour as a site of structural exploitation, but Chilli Powder, Half Sari, and Single show that Dalit women in the collection carry a burden compounded rather than simply added to by gender, consistent with what scholars describe as the double marginalisation of Dalit women, excluded by caste from many spaces and opportunities while simultaneously subordinated within their own communities by gender, the two systems reinforcing rather than merely accumulating (Pan, 2018; Mangat and Gill, 2024).

Chilli Powder contains, in the reviewers’ own assessment, the richest intersectional material in the collection, an upper caste woman deploying caste power directly against a Dalit woman, and it repays closer attention than a summary table can offer. Gangamma, feared throughout the village, throws chilli powder into Pachayamma’s face for cutting grass on her land, an act of violence Pachayamma has no institutional recourse against. Yet Pachayamma returns to the same task the following day, not from any new submission to Gangamma’s authority but because her family’s hunger remains a stronger and more immediate force than Gangamma’s cruelty. Later, arrested for taking cotton from someone else’s land, she alone among the women caught cannot afford to pay her fine and leave. What makes this story distinctively intersectional rather than simply an instance of caste conflict is that the violence against Pachayamma comes from another woman, using caste power as a weapon in a way no male character in the collection experiences, while the economic bind that puts her on that land in the first place, unable to own land through her caste position, unable to stop working because her family’s survival depends on her daily labour, is itself gendered by the fact that this labour and its risks fall to her rather than being shared or absorbed elsewhere in the household. Bama never explains this double bind through authorial commentary; she shows an empty kitchen and an arriving policeman and trusts the reader to understand what has been left unsaid (Sinha, 2024). Sinha (2024) argues that the broader agrarian system in India effectively criminalises the survival strategies Dalit women are forced to rely upon, offering no legitimate path forward while punishing whatever illegitimate path is attempted instead, and Chilli Powder is that argument rendered as a woman’s face burning in a cotton field.

Half Sari develops what Table 2 records as the collection’s starkest negative case, a story in which no form of resistance is available at all. Chellakkili’s father, Irulappan, hoping to see his daughter become a schoolteacher, sends her to a landlord’s household on the promise that she will help with light domestic work before attending school alongside the landlord’s own daughter. She is never sent to school. She works instead as an unpaid domestic servant, is never permitted the promised visits home, and eventually dies of overwork, her family learning of her death only after the funeral has already taken place. Unlike Pachayamma, who at least retains the option of returning to the field the next day, Chellakkili has no comparable margin for resistance, because the currency being exploited is not her labour alone but her body, her marriageability, and the hope her family has placed in her future, all extracted through a promise the landlord appears never to have intended to keep, calculated exploitation dressed in the outward appearance of kindness (Pan, 2018). The half sari of the title, traditionally given to a girl at puberty to mark her transition into womanhood, becomes within the story a symbol of everything promised to Chellakkili and never delivered: her education, an ordinary protected girlhood, and any future of her own choosing. That betrayal forecloses agency so completely precisely because it operates through the specifically gendered promise of mobility, a pattern of exploitation through hope that Soundararajan et al. (2023) identify as common to informal labour arrangements across rural India and that Simon (2023) argues personal narratives of this kind render visible as structural rather than incidental.

Single provides the collection’s clearest instance of a Dalit woman successfully resisting this compounded burden, and the contrast with Half Sari is instructive. Illamalli, unmarried past the age the village considers acceptable, is mocked and then physically assaulted by a man who invokes the label single the whole village already uses against her. She escapes and tells her grandmother, who tells her plainly that men of this kind will only understand once she herself takes decisive action. Word soon spreads that the landlord Naikkar has become single-eyed. Bama never describes what Illamalli did or how the landlord lost his eye, and that deliberate silence is itself meaningful: the village understands without needing it spelt out, and from that day forward nobody calls Illamalli single again. Pan (2018) writes of Dalit women who refuse the stigmatised identities caste and gender hierarchies attempt to impose on them, and Illamalli does exactly this, not through petition to any authority but through direct action whose precise nature the story withholds even from its own readers. Where Chellakkili had no resistance available to her because the exploitation ran through hope and promise rather than open confrontation, Illamalli’s resistance exists but can only survive as an unspoken, deniable act, confirming that even where Dalit women in the collection do resist successfully, the form that resistance is permitted to take differs from the public, named confrontation available to men such as Ammasi or Maruthappan elsewhere in the collection.

This same compounding logic extends beyond direct action into language itself. Pandey and Niraula’s (2024) critical discourse analysis of proverbs shows how everyday sayings encode and normalise caste hierarchy well before any single act of discrimination occurs, functioning, much as the honorifics examined in Annachi above, as a linguistic infrastructure that makes caste-based and gendered subordination feel self-evident rather than imposed. Read alongside that argument, Gangamma’s violence, the landlord’s broken promise to Chellakkili, and the label single itself are not isolated incidents, but instances of a wider discursive pattern in which caste and gender are jointly naturalised through the ordinary language a community uses about its own women, well before either system needs to resort to direct force.

To address the comparative dimension of RQ3 directly, it is worth setting this pattern against the experience of Dalit men in the same stories. Men’s experience of caste subordination in the collection is repeatedly organised around risk to the body and to labour: Madasami’s position is expressed through unequal exchange and spatial exclusion, Muthu’s through a fatal command he cannot refuse, Malandi’s through a debt that can never be repaid. In each case the threat is external and structural, and in each case some form of resistance, however small, remains available to the man facing it, whether Madasami’s eventual recognition, Malandi’s fearlessness, or Ammasi’s small refusal on the bus. Dalit women in the collection face this same structural threat, but it is compounded rather than replaced by a second axis of vulnerability rooted in their bodies, their marriageability, and their unnegotiated domestic obligations. Kaliyamma cannot simply choose to prioritise her daughter’s schooling because childcare falls to her by default rather than by negotiation between the couple. Pachayamma’s caste-based poverty is enforced through a direct physical assault from another woman, Gangamma, using caste power as a weapon in a way no male character in the collection experiences. Chellakkili’s family’s poverty is exploited specifically through the currency of her marriageability and her body’s labour, promised an education and delivered instead into unpaid domestic servitude that ends in her death. This compounding is what table 2 summarises as double marginalisation, and Crenshaw’s (1989) concept of intersectionality names precisely this structural pattern, in which caste and gender do not simply add together but interact to produce a distinct and more dangerous form of vulnerability than either alone. Chakravarti’s (1993) account of what Rege (1998) terms brahmanical patriarchy is directly relevant here, since both scholars argue that the control of caste purity has historically depended on controlling women’s bodies, sexuality, and labour specifically, which is precisely why Gangamma’s assault on Pachayamma and the marriage-linked exploitation of Chellakkili in Half Sari take the particular gendered forms that they do rather than resembling the more direct, labour-focused subordination visited on Dalit men in the collection. Guru’s (1995) related argument, that Dalit women occupy a doubly subaltern position even within Dalit political discourse itself, further explains why the women in Bama’s stories rarely have access to the kind of organised, named collective response that Maruthappan leads in Those Days.

The comparison also extends to the form that resistance itself is permitted to take. When a man organises a public response to caste-based violence, as Maruthappan does in Those Days, he gathers 20 people and confronts the perpetrator directly in the open street, and this collective, named, and visible action is treated by the community as legitimate. When a woman resists a comparable threat, as Illamalli does in Single, the same public visibility is not available to her. Her response remains unspoken and deniable, knowable only through its consequence, the landlord’s blinding, rather than through any named act the story is willing to describe directly. Read together, these two moments suggest that Dalit men and Dalit women in Bama’s fiction do not simply experience different intensities of the same oppression. They operate under different terms of engagement with it, in which men retain some access, however constrained, to public and collective response, while women’s most decisive acts of resistance are pushed further into the hidden transcript that Scott (1990) describes, unnameable even within the story that depicts them.

### 6.5 Resistance: from silence to speech

Those Days, The Ichi Tree Monkey, and Ponnuthayi, read together, mark the outer boundaries of what resistance can look like in Bama’s fiction, from its most organised and public form to its complete absence, and the distance between them is the clearest evidence the collection offers for how conditional the shift from submission to assertion actually is.

Those Days shows resistance at its most organised. The story opens by noting that lower caste villagers once stood in silence whenever a landlord passed, a ritual of deference repeated across countless ordinary days, but that growing access to education and political consciousness has begun to change this pattern. When an upper caste man sexually assaults a young Paraiyar girl, Maruthappan refuses the settlement that might once have been the only realistic option, a quiet payment or a forced marriage to the perpetrator and instead gathers around 20 people to confront the man publicly, naming the assault rather than allowing it to be quietly absorbed. Mangat and Gill (2024) document how Dalit women who experience sexual violence are routinely pressured, often by their own wider community, into accepting exactly this kind of settlement rather than pursuing accountability, which makes Maruthappan’s refusal a challenge to an entire established pattern rather than to one perpetrator alone. Bikshapathi (2021) connects this collective action to the psychological legacy of the Self Respect Movement, and the shift it represents, from unreflective acceptance of subordination to a critical recognition that the arrangement is neither natural nor inevitable, is close to what Freire (1970) calls conscientização, the process by which an oppressed group comes to perceive the contradictions in its own situation and act against them.

The Ichi Tree Monkey offers a different register of the same underlying idea. The title story follows a clever monkey that consistently outsmarts everyone who tries to catch it, and read allegorically, the monkey becomes a figure for Dalit communities themselves, small in terms of formal power, routinely underestimated, and persistently capable of finding a way through traps set by others. Jaiswal (2023) argues that folk and community narratives of this kind carry layers of cultural meaning that mainstream literary tradition has tended to dismiss as simple entertainment, and Bama’s choice of this story as her collection’s title signals that she views this kind of folk wisdom, practical cunning passed down through generations of animal and trickster tales, as central to how Dalit communities have actually managed to survive, a mode of resistance that is neither Maruthappan’s public confrontation nor Ammasi’s linguistic refusal but something closer to persistent, unglamorous evasion.

Ponnuthayi marks the point at which this same resistance simply fails to appear at all, and Table 2 records this precisely: no resistance visible, absence itself is the message. Ponnuthayi, an elderly woman who has spent her adult life in continuous unpaid service to upper caste households, moves between homes across a single representative day, completing what is expected of her without complaint and without ever asking for more, having learned across many decades that asking changes nothing. Pal (2015) describes this as one of the strongest effects of sustained social exclusion, occurring when discrimination becomes so consistent that it stops feeling imposed and starts feeling simply like life itself. What makes Ponnuthayi’s absence of resistance analytically significant, rather than merely sad, is the contrast it forms with Madasami in Pongal, whose recognition comes late but does eventually arrive, and with Maruthappan, whose recognition becomes collective action. Ponnuthayi is never shown reaching that threshold. She remains warm and genuinely loved by the children in the households where she works, not broken in any simple sense, but she has never had the opportunity to discover what else she might have become, and Bama offers no dramatic moment, no missing towel or broken promise, at which the underlying injustice finally becomes visible to her. The absence of any such moment is precisely the point: conscientização, in Freire’s (1970) sense, is not automatic or guaranteed by exposure to injustice alone, and Ponnuthayi’s quiet, undramatic story is the collection’s clearest reminder that some lives pass entirely within the public transcript Scott (1990) describes, with no hidden transcript ever surfacing at all.

### 6.6 Collective community resilience

The forms of resistance examined so far are overwhelmingly individual, even where, as in Those Days, an individual’s refusal draws 20 people into a single public confrontation. The Ancharamanippoo Tree describes a different register of resistance again, one that is collective from the outset rather than collective because one person’s action recruits others to it. The story centres on a sacred tree that lower caste communities have long used for festivals, rituals, and gatherings. When upper caste landlords attempt to restrict access to the tree and the land surrounding it, explicitly asserting ownership over a space the community has used across generations, the community’s response is not organised around a single leader confronting a single perpetrator, in the way Maruthappan confronts the assailant in Those Days. It is instead a standing, pre-existing claim that the community simply continues to assert, gathering at the tree as they always have, treating the landlord’s attempted restriction as illegitimate rather than as a threat requiring a dramatic act of defiance to answer. Bikshapathi (2021) traces how the Self Respect Movement encouraged Dalit communities across Tamil Nadu to reclaim their own religious and cultural practices from beneath the dominance of Brahminical tradition, and this story enacts that reclamation not as an argued position but as a simple, sustained action, a community returning to a tree that has always been theirs.

Read alongside Those Days, The Ancharamanippoo Tree shows that collective resilience in Bama’s fiction takes at least two distinct forms. One is reactive and escalating: an individual injustice provokes an individual response that grows, through deliberate organising, into a collective one, as when Maruthappan’s refusal becomes a march of 20 people to the landlord street. The other is standing and continuous: a claim to shared space and shared practice that the community maintains simply by continuing to exercise it, without needing to be provoked into being reasserted. Both forms depend on the same underlying shift Bikshapathi (2021) associates with the Self Respect Movement, from treating subordination as natural to treating it as a constructed arrangement that can be refused, but they differ in what triggers the collective response and in how visible the moment of resistance is to an outside observer. A reader looking for resistance only in the form of a confrontation, a march, or a named act of defiance would find The Ancharamanippoo Tree comparatively quiet, in much the way Ponnuthayi’s absence of resistance could be mistaken for its opposite. What the story in fact demonstrates is that some forms of Dalit collective resilience are not events at all, but a sustained refusal to relinquish a claim, made visible only in its persistence.

## 7 Conclusion

Bama’s *The Ichi Tree Monkey* is not simply a collection of short stories meant for casual reading. Read carefully and as a whole, it functions as a genuine cultural psychological document in its own right. It shows with real precision how caste operates directly on the human mind, shaping identity from a very early age, steadily limiting what a person believes is genuinely possible for themselves, and producing, often within the very same individual communities and even within the very same families, both deep submission and quiet, persistent resistance existing side by side rather than as opposites that can never coexist.

The 15 stories examined closely across this paper show that Bama’s Dalit characters tend to move, slowly, unevenly, and frequently at considerable personal cost, away from a position of conditioned acceptance and toward a more conscious and deliberate refusal of the conditions imposed upon them. This particular movement is never presented by Bama as automatic, guaranteed, or simple. Some characters across the collection never manage to complete this movement at all within the timeframe of their own individual story, remaining caught instead within patterns of submission that were established long before the story itself even begins. Yet some genuine trace of this underlying movement, however small or incomplete, is present in nearly every story examined here, and it remains, throughout, recognisably and consistently human in its texture and detail rather than abstract or purely theoretical.

It is worth pausing here to consider what this movement actually looks like when it does appear, since it rarely resembles the kind of dramatic transformation that popular narratives about resistance often lead readers to expect. Scott (1985, 1990) makes this point precisely about subordinate groups more broadly, arguing that everyday resistance is deliberately unspectacular because visibility itself carries risk, and that the more revealing story is usually told in small, plausibly deniable acts rather than open confrontation. Madasami does not deliver a speech. He simply walks home one evening and, for the first time, allows himself to notice what he has been refusing to notice for years. Ammasi does not organise a movement. He simply keeps his seat on a bus and calls a man by an ordinary word. Illamalli does not file a complaint with any formal authority. She takes action herself, in the only language that has ever produced results within her own village. Maruthappan, admittedly, does organise something closer to a recognisable collective response, gathering 20 people and walking to the landlord street together, and this difference matters, because it shows that the movement from submission toward resistance can take both modest individual forms and more visibly collective ones, sometimes within the very same small community and even within the same extended family across different generations. What unites all of these moments, regardless of their scale, is that each one represents a small but real refusal to accept, without question, an arrangement that had previously been accepted without question for a very long time.

For the discipline of cultural psychology specifically, Bama’s fiction offers something genuinely important and not easily replicated through other research methods. It offers clear, detailed, and emotionally grounded evidence that psychological resilience within marginalised communities is not best understood as a simple, fixed personality trait that some individuals happen to possess and others unfortunately lack. It is instead best understood as a social and cultural achievement, one that is built gradually over time through community solidarity, through a developing sense of historical consciousness about one’s own community’s past, and through the steady accumulation of small, everyday refusals to simply accept whatever has been imposed from outside. Malandi Thatha’s clear eyed refusal to be diminished by his own bonded debt, Arayi’s single afternoon of genuine laughter, and Maruthappan’s public confrontation in the landlord street are not isolated, unconnected incidents scattered randomly across the collection. They are, instead, different expressions of the very same underlying psychological capacity, a capacity that Bama shows us is cultivated collectively across an entire community over a long period of time, rather than simply inherited individually by a fortunate few.

This raises a further question of significance beyond the immediate scope of the three research questions above, namely whether this psychological logic travels beyond the specific context of caste in Tamil Nadu. Caste is a system particular to South Asia, and this paper does not claim that Dalit experience is equivalent to, or interchangeable with, other forms of structural discrimination elsewhere. What does appear to travel, however, is the underlying mechanism of resistance itself. Kelley (1994) documents a strikingly similar pattern among the Black working class in the Jim Crow era United States, drawing on the same Scott (1985, 1990) framework applied here, to show how racial discrimination produced its own repertoire of quiet, low-visibility, and often deniable acts of resistance, from feigned deference on the factory floor to loud talk on a streetcar, precisely because open confrontation with white authority carried lethal risk. The specific content of that resistance was shaped by race rather than caste, and it was built inside a different history entirely, but the underlying logic, that subordinate groups develop small, cumulative, and plausibly deniable forms of refusal wherever open confrontation is too dangerous, is the same logic this paper has traced through Bama’s fiction. Read this way, Bama’s characters are not offered here as evidence that caste and race are the same thing, but as a further, richly detailed instance of a psychological pattern that recurs wherever discrimination is structural, sustained, and dangerous enough to resist openly.

This paper has also tried to show, through sustained and detailed close reading across all 15 stories rather than a brief or partial selection, that Bama’s short fiction rewards exactly this kind of patient psychological attention. Each story, examined carefully on its own terms, reveals a different facet of how caste-based oppression actually operates on the inner lives of the people subjected to it, and each story simultaneously reveals a different facet of how those same people find ways, however small or however costly, to hold onto a meaningful sense of themselves regardless. Read individually, these stories function as moving, self-contained pieces of literary fiction. Read together, as this paper has attempted to read them, they begin to function as something closer to a sustained psychological argument about an entire community, made not through abstract theory or statistical measurement, but through the accumulated weight of 15 carefully observed human lives.

Simon (2023) argues persuasively that Dalit women’s writing of this general kind consistently transforms individual personal testimony into a broader form of collective political evidence, evidence that can then be examined, cited, and taken seriously well beyond the immediate community in which it originated. This shift echoes Paik’s (2018) broader account of the rise of new Dalit women within Indian historiography, in which women who were once written about are increasingly recognised as subjects and authors of their own historical and literary record. Bama’s 15 stories accomplish exactly this transformation, and they do so with a level of craft, restraint, and emotional precision that places them firmly within serious literature, while simultaneously functioning as a genuine record of psychological experience under sustained caste-based oppression. They are not simply literature in the narrow, purely aesthetic sense of that word. They are also, at the very same time, a record, carefully kept and carefully shared, of what caste actually does to people across an entire lifetime, and equally, of what people themselves consistently choose to do in return, even when that choice is small, quiet, and visible only to those paying very close attention.
